# D-Carvone Attenuates CCl_4_-Induced Liver Fibrosis in Rats by Inhibiting Oxidative Stress and TGF-ß 1/SMAD3 Signaling Pathway

**DOI:** 10.3390/biology11050739

**Published:** 2022-05-12

**Authors:** Hanan A. Ogaly, Sharah A. A. Aldulmani, Fatimah A. M. Al-Zahrani, Reham M. Abd-Elsalam

**Affiliations:** 1 Department of Chemistry, College of Science, King Khalid University, Abha 61421, Saudi Arabia; saldolmani@kku.edu.sa (S.A.A.A.); falzhrani@kku.edu.sa (F.A.M.A.-Z.); 2 Department of Biochemistry, Faculty of Veterinary Medicine, Cairo University, Giza 12211, Egypt; 3 Department of Pathology, Faculty of Veterinary Medicine, Cairo University, Giza 12211, Egypt

**Keywords:** D-carvone, fibrosis, antioxidant, α-SMA, TGF-β1/SMAD3, MMP9

## Abstract

**Simple Summary:**

Liver fibrosis is a challenging global health problem resulting in a significant morbidity and mortality rates worldwide due to its rapid progression to cirrhosis and hepatocellular carcinoma. Therefore, identifying nontoxic therapies with precise curative effects to slow the progression of liver fibrosis comprises one of the most popular and high-priority areas of current research. D-carvone is a naturally occurring monoterpene abundant in the essential oil of aromatic plants such as caraway and spearmint. In the present study, the protective impact of D-carvone on carbon tetrachloride (CCl_4_)-induced liver fibrosis in rats was evaluated. Administration of D-carvone significantly enhanced liver functions, oxidant/antioxidant balance as well as liver histology. D-carvone ameliorated the progression of liver fibrosis, evident by the decreased collagen deposition (fibrosis score) and the reduced expression of the pro-fibrogenic markers TGF-β1 and SMAD3 in the liver. These findings reveal the anti-fibrotic effects of D-carvone and suggest that D-carvone could be a promising candidate for therapeutic intervention of liver fibrosis and other oxidative stress-related hepatic diseases.

**Abstract:**

D-carvone is a natural monoterpene found in abundance in the essential oil of aromatic medicinal plants with a wide range of pharmacological values. However, the impact of D-carvone on liver fibrosis remains unclear. This study aimed to evaluate the anti-fibrotic potential of D-carvone in a rat model of liver fibrosis and to clarify the possible underlying mechanisms. Liver fibrosis was induced in rats by carbon tetrachloride, CCl_4_ (2.5 mL/kg, interperitoneally every 72 h for 8 weeks). Oral treatment of rats with D-carvone (50 mg/kg, daily) started on the 3rd week of CCl_4_ administration. D-carvone significantly enhanced liver functions (ALT, AST), oxidant/antioxidant status (MDA, SOD, GSH, total antioxidant capacity; TAC), as well as histopathological changes. Moreover, D-carvone effectively attenuated the progression of liver fibrosis, evident by the decreased collagen deposition and fibrosis score by Masson trichrome staining (MT) and α-SMA protein expression. Moreover, D-carvone administration resulted in a significant downregulation of the pro-fibrogenic markers TGF-β1 and SMAD3 and upregulation of MMP9. These findings reveal the anti-fibrotic effect of D-carvone and suggest regulation of the TGF-β1/SMAD3 pathway, together with the antioxidant activity as a mechanistic cassette, underlines this effect. Therefore, D-carvone could be a viable candidate for inhibiting liver fibrosis and other oxidative stress-related hepatic diseases. Clinical studies to support our hypothesis are warranted.

## 1. Introduction

Liver fibrosis is a challenging global health problem resulting in a significant percent of morbidity and mortality worldwide [1]. Despite being a reversible wound-healing response to various chronic liver injuries, liver fibrosis could range in its pathological spectrum from defined depositions of extracellular matrix (ECM) to cirrhosis that may result in hepatocellular carcinoma [2]. Following sustained liver injury in response to chemokines, cytokines, and other noxious stimuli, a series of pathological events take place leading to the activation of resident mesenchymal cells called hepatic stellate cells (HSCs) found in the subendothelial space of Disse. Activation of HSCs leads to their trans-differentiation from a quiescent to an active, proliferative and contractile myofibroblast-like cell [3]. These, in turn, over-express the cytoskeleton protein, α-smooth muscle actin (α-SMA), and disrupt the matrix metalloproteinase (MMP) homeostasis resulting in massive non-functioning ECM, the key driving factor implicated in the fibrotic process [4].

Numerous studies on the pharmacotherapy of hepatic fibrosis have been emerged. Strategies of prevention and treatment of liver fibrosis, according to their mechanism, are directed mainly to inhibit HSCs activation, inhibit ECM production, promote ECM degradation, and induce activated HSCs apoptosis [5]. However, due to the complex pathophysiological mechanism of liver fibrosis, most of the existing therapies fail to meet the necessary requirements, and thus few of them have been clinically applied [6]. Therefore, identifying nontoxic therapies with precise curative effects to slow the progression of liver fibrosis comprises one of the most popular and high-priority areas of current research [2].

Currently, an increasing number of reports have noticed the pivotal role of oxidative stress in the pathogenesis of liver fibrosis. Therefore, importance was specified to antioxidant as the potential solution. In this context, several lines of evidence have suggested the anti-fibrotic activities of a variety of natural phytochemicals [7,8]. Carvone (p-mentha-6,8-dien-2-one) is a natural unsaturated monoterpene found as a major constituent in the essential oil of some aromatic medicinal plants, such as caraway (*Carum carvi* L.) and spearmint (Mentha spicata) [9,10]. Carvone constitutes a common ingredient in the human diet, as it is widely used as a flavor and odorant accompaniment and a known folk medication for gastric disorders and diarrhea [11]. Two enantiomeric forms of carvone (S- and R-) have been identified that vary in their pharmaceutical values. This variation may be explained by the Stereoselective metabolism of carvone enantiomers by the liver microsome [12]. D-carvone enantiomer has shown a wide range of pharmacological effects including immunomodulatory, anti-tumorigenic, chemo-preventive, anti-hyperlipidemic, and anti-hypertensive [9,13,14,15,16,17]. The hepatoprotective effects of carvone were previously reported against immobilization-induced oxidative liver injury [18]. Nevertheless, to our knowledge, no previous studies have assessed the role of D-carvone on liver fibrosis and its underlying molecular mechanism.

Based on a number of in vivo and in vitro studies, various mechanisms are proposed to explain the biomedical actions of D-carvone in health and diseases. A considerable extent of the pharmacological activities of D-carvone is attributed to its antioxidant properties [19]. It has been suggested that the general mechanism of action of D-carvone is through induction of glutathione S-transferase (GST) [20]. GST is a phase II detoxifying enzyme that catalyzes the conjugation of glutathione (GSH) to a wide variety of oxidants via speeding up the trapping of electrophilic groups present in various substrates by the thiolate group of GSH [21]. D-carvone acts also by enhancing the activity of other endogenous antioxidants such as GSH, glutathione reductase (GR), and glutathione peroxidase (GPx), contributing to the cellular antioxidant defense mechanism [19]. Moreover, D-carvone and its derivatives appear to target multiple signaling pathways related to inflammation and oxidative stress including NF-κB pathway [18], nuclear factor erythroid 2-related factor 2 (Nrf2) pathway [22], Janus kinase (JAK)/signal transducer and activator of transcription 3 (STAT3) signaling pathway [23], and nucleotide-binding oligomerization the domain-like receptor 3 (NLR3)/transmembrane toll-like receptor 4 (TLR4) cascade [24]. Since inflammation, oxidative stress, and fibrogenesis are intrinsically linked, we hypothesize that D-carvone could exhibit an anti-fibrotic action as a consequence of its anti-inflammatory and antioxidant effects [18,24,25]. Herein, the current study aimed to inspect the anti-fibrotic potential of D-carvone on CCl_4_-instigated liver fibrosis in rats and its efficiency to modulate some pro-fibrogenic and HSCs activating proteins as a mechanism contributing to its anti-fibrotic mechanism.

## 2. Results

### 2.1. Effect of D-Carvone on Liver Function Indices in CCl_4_-Induced Liver Fibrosis

**Figure 1:**
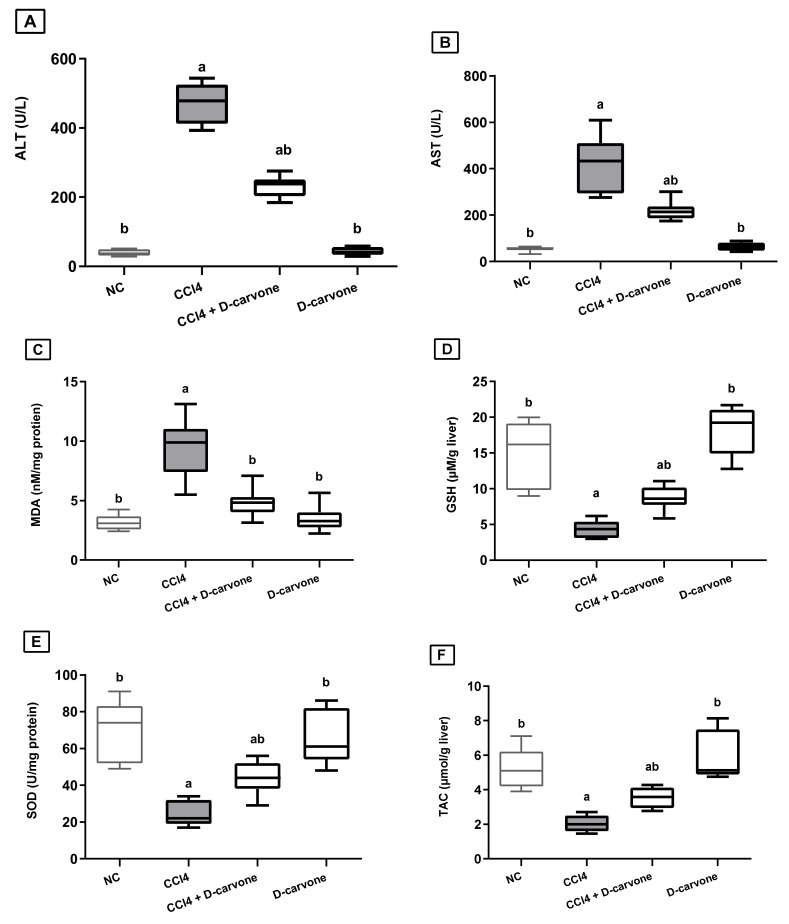
Effect of D-carvone on serum and liver biomarkers in CCl_4_-intoxicated rats. (**A**) serum ALT, (**B**) serum AST, (**C**) liver MDA, (**D**) liver SOD, (**E**) liver GSH, and (**F**) liver TAC. Values are expressed as mean ± SD (*n* = 7). Data were analyzed by one way ANOVA followed by Tukey’s test, ^a^ *p* ≤ 0.05 compared with NC and ^b^ *p* ≤ 0.05 compared with CCl_4_ group. NC, normal control; CCl_4_, carbon tetrachloride; ALT, alanine aminotransferase; AST, aspartate aminotransferase; MDA, malondialdehyde; SOD, superoxide dismutase; GSH, glutathione.

Figure 1 confirmed the severe hepatic injury in the fibrosis model, where in the CCl_4_-intoxicated group, the serum levels of ALT and AST were elevated by 12.5- and 7.3-fold, respectively, as compared to the normal control, while administration with D-carvone (50 mg/kg, bw) significantly reduced serum ALT and AST to 54.8% and 51% compared to the CCl_4_ model group, respectively (Figure 1A,B).

### 2.2. Effect of D-Carvone on Liver Oxidant/Antioxidant Biomarkers in CCl_4_-Induced Liver Fibrosis

As shown in Figure 1, injection of CCl_4_ resulted in a significant elevation in MDA content (2.9-fold) and a significant depletion in GSH content (0.19-fold) and SOD activity (0.33-fold) with a significant reduction in TAC in the liver, as compared to normal group values. Treatment with D-carvone (50 mg/kg), two weeks after induction of hepatic injury, effectively decreased the MDA levels to 50.7%, while it restored the depleted GSH and SOD levels to 233% and 221.5%, respectively, as compared to CCl_4_ control group (Figure 1C–E). Moreover, D-carvone significantly restored the hepatic TAC as compared to CCl_4_ control group (Figure 1F).

### 2.3. Effect of D-Carvone on Liver Histopathological Changes in CCl_4_-Induced Liver Fibrosis

**Figure 2:**
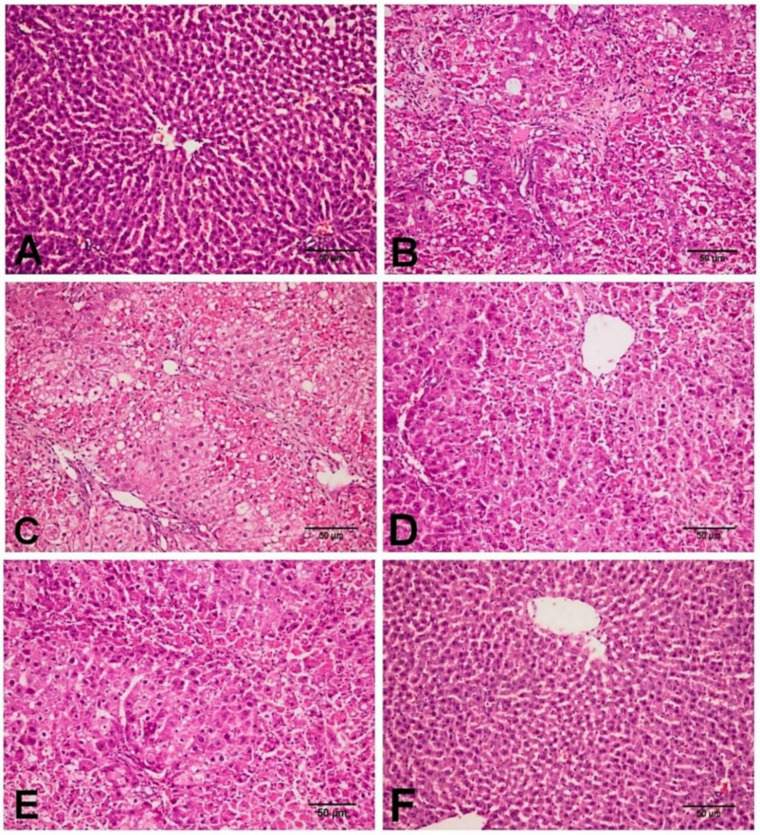
Photomicrograph of the liver tissues in the different experimental group. (**A**) NC group showing normal histological architecture of the liver with normal hepatic cords and hepatocytes. (**B**,**C**) CCl_4_ group showing hepatocellular necrosis in periportal and pericentral areas with macrovesicular and microvesicular steatosis, mononuclear inflammatory cell aggregation, and marked collagen fibers proliferation with disorganization of hepatic cords. (**D**,**E**) CCl_4_+ D-carvone group showing moderate hepatocellular necrosis around the central vein with mild mononuclear inflammatory cells infiltration. (**F**) D-carvone group showing normal hepatic cellular findings.

As shown in Figure 2, the histopathological examination of the control as well as the D-carvone group revealed normal hepatic architecture with normally arranged hepatic cords (Figure 2A,F). On the one hand, the CCl_4_ treated group revealed severe hepatic lesions in the form of hepatocellular necrosis in periportal and pericentral areas associated with mononuclear inflammatory cell aggregations, macrovesicular, and microvesicular steatosis, and marked collagen fibers bridging and disorganization of hepatic cords (Figure 2B,C). On the other hand, the group treated with D-carvone showed marked enhancement in the hepatic lesions, and the liver revealed mild to moderate hepatocellular necrosis with mild inflammatory cell aggregations (Figure 2D,E).

### 2.4. Effect of D-Carvone on Collagen Deposition in Liver of CCl_4_-Induced Liver Fibrosis

**Figure 3:**
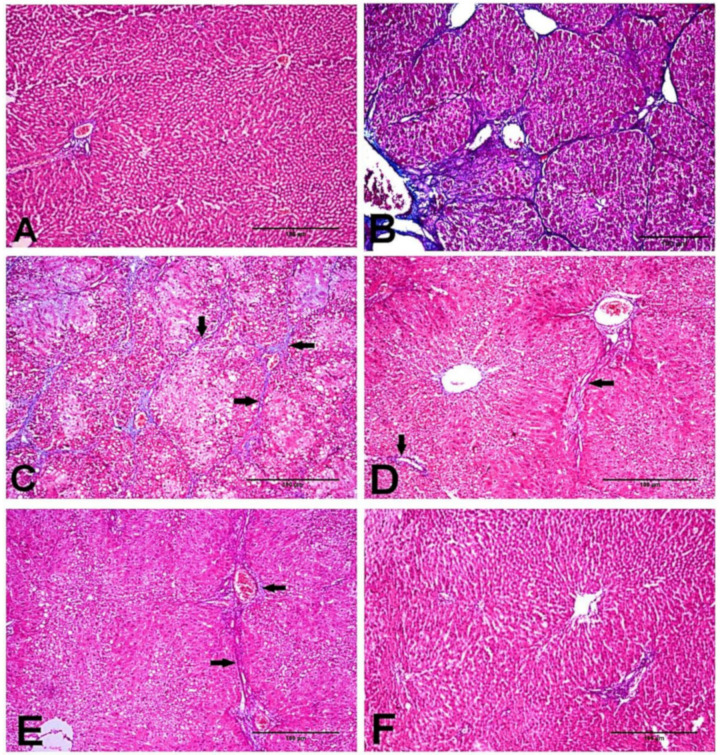
Photomicrograph of the liver tissues stained with MT stain. (**A**) NC group showing normal deposition of collagenous fibers in the portal areas. (**B**,**C**) CCl_4_ group showing marked collagenous fibrous bridging with excessive collagen fibers deposition from portal to central areas and from central to central areas grade 5 (arrows). (**D**,**E**) CCl_4_+ D-carvone group showing marked diminution of collagen fibers deposition and distribution from portal to portal areas. (**F**) D-carvone group showing normal collagen fibers spreading around portal area.

**Figure 4:**
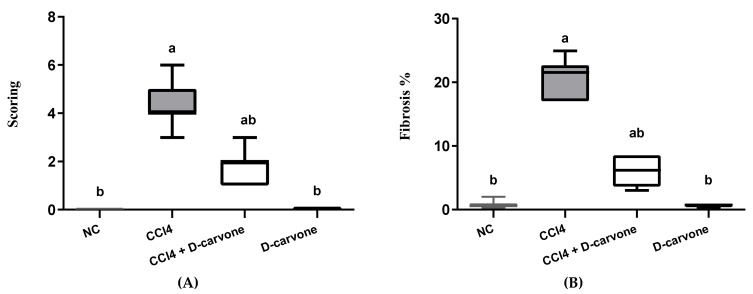
Effects of D-carvone on liver fibrosis scoring and liver fibrosis % in CCl_4_-intoxicated rats. (**A**) Liver fibrosis score system. (**B**) Liver fibrosis %. Values are expressed as mean ± SD (*n* = 7). Data were analyzed by one way ANOVA followed by Tukey’s test, ^a^ *p* ≤ 0.05 compared with NC and ^b^ *p* ≤ 0.05 compared with CCl_4_ group.

As shown in Figure 3, the tissue section stained with MT stain showed the distribution of collagen fibers in the different groups. NC and D-carvone treated groups revealed normal distribution of collagen fibers as in the periportal areas (Figure 3A,F). To begin with, CCl_4_ group showed abundant collagen fibers around the portal areas, collagen fibers bridging among portal areas, and portal to central and central to central areas (Figure 3B,C). In addition, liver fibrosis percent as well as liver fibrosis score was significantly elevated in CCl_4_ compared to the normal group (Figure 4). Nevertheless, the group treated with D-carvone showed a significant decline in both liver fibrosis score (Figure 4A) and liver fibrosis percent (Figure 4B), and their collagenous septa became thinner than those that were observed in the CCl_4_ group and recorded only around the portal areas and from portal-to-portal areas (Figure 3D,E).

### 2.5. Effect of D-Carvone on TGF-β and MMP9 Genes Expression

**Figure 5:**
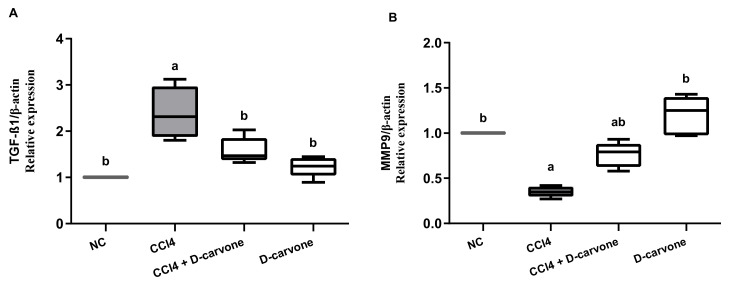
Effects of D-carvone on the expression of TGF-β and MMP9 transcription levels in liver tissues CCl_4_-intoxicated rats. The transcription levels of TGF-β (**A**) and MMP9 (**B**) genes in the hepatic tissues. The relative mRNA expression is represented as fold change over the normal control value after normalization to β-actin using the 2^−ΔΔCT^ calculation method. Values are expressed as mean ± SD (*n* = 5). Data were analyzed by one way ANOVA followed by Tukey’s test, ^a^ *p* ≤ 0.05 compared with NC and ^b^ *p* ≤ 0.05 compared with CCl_4_ group. NC, normal control; CCl_4_, carbon tetrachloride; TGF-β1, transforming growth factor-β1; MMP9; matrix metalloprotease 9.

Induction of hepatic fibrosis in rats using CCl_4_ resulted in a significant elevation in the pro-fibrogenic marker TGF-β expression (2.45-fold) as compared to normal control value (Figure 5A). Treatment of CCl_4_-intoxicated rats with D-carvone 50 mg/kg resulted in significantly downregulated hepatic TGF-β expression (1.54-fold) as compared to CCl_4_ group (2.45-fold). On the other hand, CCl_4_ intoxication resulted in a significant reduction in hepatic MMP9 to 0.36-fold as compared to normal control values. D-carvone significantly restored MMP9 expression to 0.76-fold (Figure 5B).

### 2.6. Effect of D-Carvone on Hepatic α-SMA, TGF-β1, and SMAD3 Protein Expression

**Figure 6:**
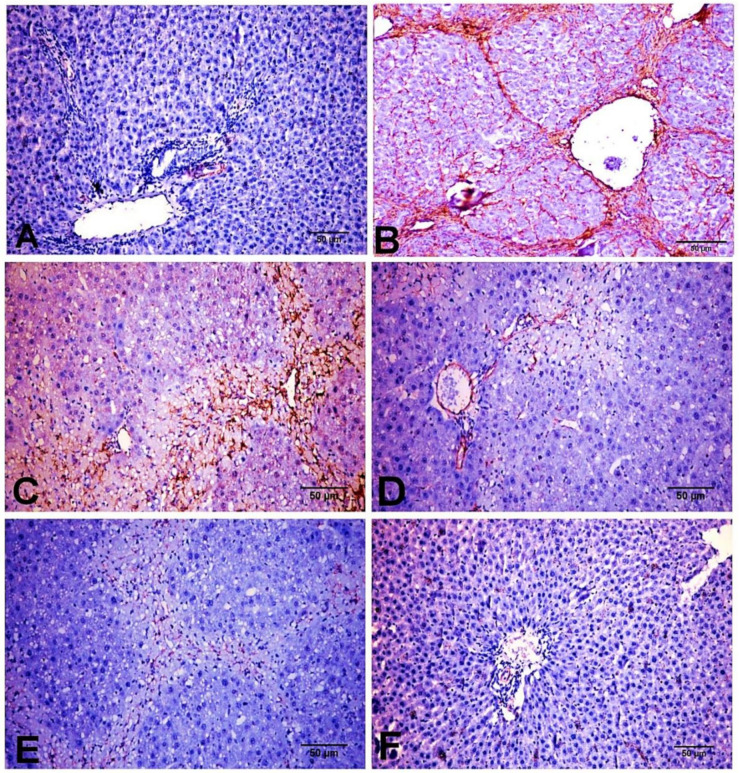
Photomicrograph of α-SMA expression in the different experimental groups. (**A**) NC group. (**B**,**C**) CCl_4_ group showing strong immunostaining reaction in spindle shape myofibroblast cells along collagenous fibrous bridging. (**D**,**E**) CCl_4_+ D-carvone group showing marked reduction in positive immunostaining myofibroblast cells. (**F**) D-carvone group.

As depicted in Figure 6, immunohistochemical analysis showed that both NC and D-carvone groups showed normal α-SMA protein expression in the smooth muscle cells of the hepatic blood vessels (Figure 6A,F). The chronic CCl_4_ intoxicated group showed significant increase in α-SMA protein expression in myofibroblast cells, which are located along collagenous septa extending from portal to portal, portal to central, and central to central areas (Figure 6B,C), whilst the D-carvone treated group exhibited a significant decline in α-SMA expression when compared with control group (Figure 6D,E).

**Figure 7:**
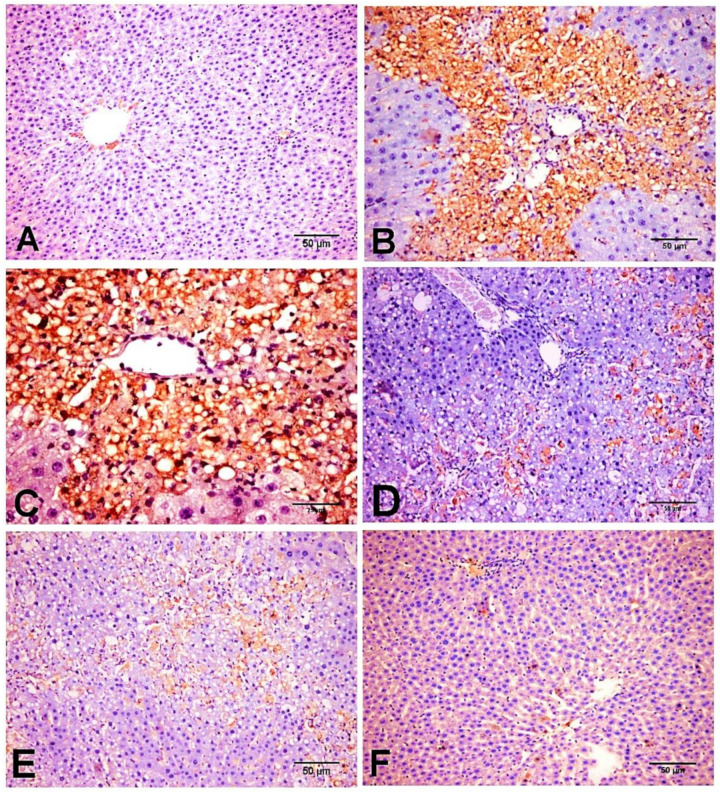
Photomicrograph of TGF-β1 expression in the different experimental groups. (**A**) NC group. (**B**,**C**) CCl_4_ group showing strong immunostaining reaction in periductal cells in the portal tract, perisinusoidal cells, around the blood vessels, sinusoidal lining cells, non-parenchymal mesenchymal cells, Kupffer cells, inflammatory cells, and hepatocytes along collagenous fibrous. (**D**,**E**) CCl_4_+ D-carvone group showing marked decline of positive immunostaining cells. (**F**) D-carvone group.

On the other hand, protein expression of the pro-fibrogenic marker TGF-β1 was markedly induced in CCl_4_ group and showed increased expression in cholingeocytes, periductal cells in the portal area, non-parenchymal mesenchymal cells, Kupffer cells, inflammatory cells, around the blood vessels, and sinusoidal lining cells, and weak to moderate expression was observed in necrotic hepatocytes along collagen bridges (Figure 7B,C), while NC and D-carvone groups showed very weak positive immunoreactivity in cholingeocyte lined bile ducts and periductal cells (Figure 7A,F). Interestingly, the group treated with D-carvone showed a significant reduction in TGF-β1 expression in livers of rats when compared with CCl_4_ treated group (Figure 7D,E).

**Figure 8:**
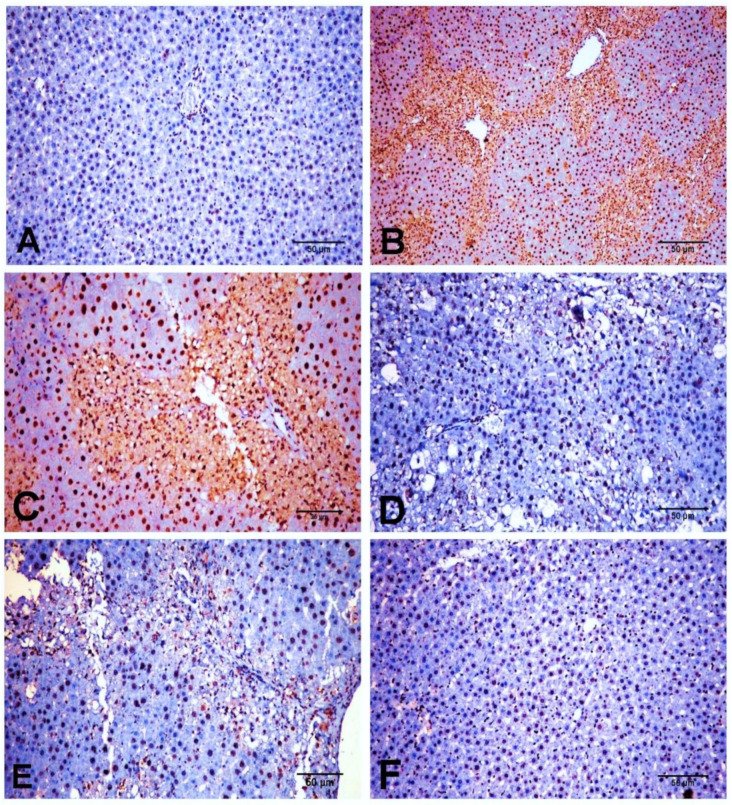
Photomicrograph of SMAD3 expression in the different experimental groups. (**A**) NC group. (**B**,**C**) CCl_4_ group showing a strong nuclear immunostaining reaction. (**D**,**E**) CCl_4_+ D-carvone group showing marked decline of positive nuclear immunostaining cells. (**F**) D-carvone group.

Similarly, SMAD3 protein expression was minimal in NC and D-carvone groups (Figure 8A,F). The CCl_4_ group exhibited a significant elevation in the nuclear expression of SMAD3 compared to the control group (Figure 8B,C), while the D-carvone treated group showed a significant decline in nuclear expression of SMAD3 compared to CCl_4_ group (Figure 8D,E).

**Figure 9:**
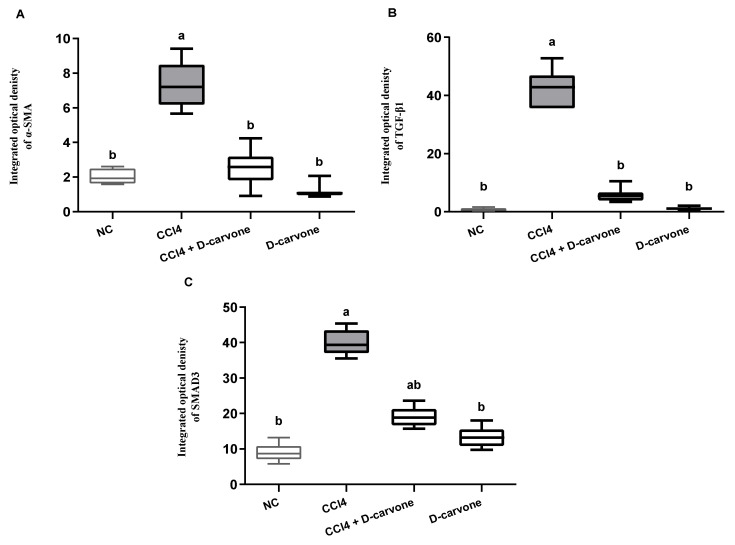
Immunohistochemical analysis of α-SMA, TGF-β1, and SMAD3 expression in the different experimental groups. (**A**) Integrated optical density of α-SMA expressed as positive area %. (**B**) Integrated optical density of TGF-β1 expressed as positive area %. (**C**) Integrated optical density of SMAD3 expressed as positive area %. Values are expressed as mean ± SD (*n* = 7). Data were analyzed by one way ANOVA followed by Tukey’s test, ^a^ *p* ≤ 0.05 compared with NC and ^b^ *p* ≤ 0.05 compared with CCl_4_ group.

These findings were further confirmed by the integrated optical density of α-SMA, TGF-β1, and SAMD3, as shown in Figure 9A–C, respectively.

## 3. Discussion

Hepatic fibrosis is the gateway to various chronic liver injuries that ultimately progress to organ failure [2]. Despite the long history of this ailment, drugs that specifically target the pathogenesis of hepatic fibrosis are rare, with side effects that restrict their use [26]. Therefore, new alternative therapeutic options are urgently needed. Recently, several experimental findings and even preliminary clinical trials have demonstrated that natural products exert beneficial therapeutic effects against fibrosis in multiple organs [27]. D-carvone is a natural plant-derived monoterpene displaying multiple pharmacological effects that are ascribed mainly to its antioxidant and anti-inflammatory effects [19,24,28]. However, its protective potential against liver fibrosis has not been investigated yet. The present study introduced novel evidence on the antifibrotic effects of D-carvone and pointed to the involvement of the antioxidative activity and TGF-β1/SMAD3 signaling modulation in mediating its effect in ameliorating fibrogenesis in CCl_4_ rat model.

CCl_4_ is a potent hepatotoxin commonly used to induce experimental liver injury, fibrosis/cirrhosis model, and chemical hepatitis [29,30]. CCl_4_ chronic administration leads liver damage due to multi-pathways including inflammation and excessive reactive oxygen species (ROS) generation along with activation and proliferation of HSCs. These events, in turn, exacerbate to hepatic fibrosis in the prolonged exposure [31]. The mechanism of CCl_4_ toxicity is attributed to the damaging effects exerted by the highly toxic CCl_4_-derived free radicals, trichloromethyl (•CCl_3_) and trichloromethyl peroxyl (•CCl_3_O_2_), which covalently bind to the cellular biomolecules and initiate a series of responses resulting in the deterioration of the hepatocellular membrane components and disruption of the protein synthesis and cellular energy process [32]. Both hepatic parenchyma and non-parenchyma cells are highly vulnerable to oxidative stress but show different responses [33]. Oxidative and nitrosative stresses cause damage of liver parenchyma cells with changes in extra cellular matrix leading to recruitment of inflammatory and immune cells to the site of injury, that in turn activate non-parenchyma cells such as HSCs and Kupffer cells [34].

Previous studies demonstrated that D-carvone displays a dose-dependent manner for its antioxidant [19], anti-inflammatory [35], hypoglycemic [36], and immunomodulatory effects [37]. Among the previously studied doses, the 50 mg/kg has been reported as a minimum effective dose [25,36,37]. In current study, we used this determined effective dose to investigate whether D-carvone has antifibrotic effect in in CCl_4_ rat liver fibrosis model. 

In the present study, the hepatoprotective and anti-fibrotic effects of D-carvone were mirrored on the improved liver indices and liver histology. Repeated CCl_4_ administration (2.5 mL/kg, for 8 weeks) significantly increased the serum ALT and AST levels indicating hepatocellular membrane damage and leakage [30,38], whereas coadministration of D-carvone (50 mg/kg) leveled these enzymes to signify its hepatoprotective effects. Similar results for the hepatoprotective effects of D-carvone on liver enzymes were previously reported [18,36].

A growing body of evidence indicates that significant impairment of the redox homeostasis is a key event for the progress of fibrogenesis in several chronic liver injuries [3,39,40]. Lipid peroxidation (LPO) and exhaustion of endogenous antioxidants have been recognized as the hallmarks of chronic CCl_4_ intoxication [41,42]. The current study illustrated similar findings where the chronic administration of CCl_4_ for 8 weeks significantly increased intrahepatic production of MDA accompanied with significant reduction in GSH content, SOD activity, and the liver TAC. Our findings showed that D-carvone relieved CCl_4_-induced liver fibrosis by ameliorating oxidative stress, as demonstrated by the significant reduction in MDA and increases in GSH, SOD and TAC in the D-carvone treated group. Evidence from previous studies indicated that D-carvone possesses potent antioxidative and free radical scavenging activities [19]. Mechanistically, D-carvone and its related compounds confer their antioxidant activities by scavenging free radicals, restoring endogenous antioxidants, and inhibiting lipid peroxidation [20,35]. D-carvone has been shown to induce GST, which is considered to be a major mechanism mediating the protective effect of D-carvone against oxidative agents and carcinogens [20]. D-carvone has been found to restore GSH level possibly by stimulating GR enzyme responsible for reduction in GSSG and maintaining GSH level [8]. GSH, beside its direct scavenging activity against a variety of reactive radicals, is also an essential factor for the antioxidant enzyme GPx, which is responsible for scavenging of H_2_O_2_ radicals [3]. In addition, D-carvone was reported to restore elements such as vitamin C and vitamin E, indicating that D-carvone showed strong antioxidative activity [19].

As previously reported, D-carvone promotes the Nrf2 signaling pathway which might contribute to the antioxidative activity of D-carvone [22]. Furthermore, it has been reported that during the metabolic pathway in the liver microsomes, (4S)-(+)-carvone is subjected to a stereoselective biotransformation into (4S,6S)-(+)-carveol [12]. Carveol could be partially attributed to the free radical scavenging properties of D-carvone. Owning to its small molecular size, carveol is able to target the Keap1/Nrf2 pathway through a preferential orientation into the shallow Nrf2-binding site of Keap1, since either upregulating antioxidant activity or suppressing lipid peroxidation was regulated by Nrf2 [43]. Therefore, activation of the Nrf2 pathway in hepatocytes is associated with reduction in oxidative stress, inflammatory response, and, correspondingly, less tissue damage and fibrogenesis [22].

On the other hand, our histopathological findings using H&E and MT revealed that CCl_4_ exposure adversely aggravated the liver homeostasis and triggered fibrogenesis. D-carvone ameliorated these histopathological alterations with a significant reduction in the fibrosis score in the liver sections. The ameliorative effect of D-carvone on progression of liver fibrosis was further confirmed by its effect on the expression of α-SMA protein, an important biomarker of HSCs activation. The intensity of α-SMA immunostaining was significantly increased in the CCl_4_ model group compared to the normal control, indicating an increase in collagen content. These results were in parallel with previous reports [44,45]. D-carvone treatment markedly reduced α-SMA producing cells, indicating inhibition of HSCs and their proliferation and regression of liver fibrosis.

D-carvone administration appreciably reduced the inflammatory cells’ infiltration and pro-inflammatory modulators release provoked by liver injury [18], ulcerative Colitis [46], and osteoarthritic condition [35]. The mechanisms mediating these ant-inflammatory effects of D-carvone include downregulation of NF-κB [18]. It appears that NF-κB, as a central protein transcription factor, can aggravate liver fibrosis through induction of inflammatory cytokines [35]. Indeed, targeting NF-κB by D-carvone could support the antifibrotic activity of D-carvone recorded in the present study.

Moreover, D-carvone has been reported to diminish the excessively produced NO by macrophages and Kupffer cells in the injured liver [18]. NO reacts with ROS producing a more reactive radical peroxynitrite (ONOO−), which can further induce HSC’s proliferation with exacerbated ECM production. These data also support the anti-fibrogenic action of D-carvone.

In addition, carvone has been found to activate the cyclic adenosine monophosphate (cAMP) signaling pathway and significantly increased cAMP levels in melanoma cells [47]. cAMP is a critical second messenger molecule that plays a key role in multiple intracellular processes in various tissues, including the liver [48]. Increasing cAMP has been found to inhibit formation and proliferation of the profibrogenic myofibroblasts and inhibit the synthesis of ECM protein in injured tissue. Hence, upregulation of the hepatic cAMP pathway could provide a potential therapeutic target to blunt liver fibrosis [49]. This evidence could suggest the contribution of raising cAMP by carvone in its anti-fibrogenic effect observed in the current study.

The current study revealed that D-carvone treatment inhibited TGF-β1 mRNA and protein expression. TGF-β1 is known as a key pleiotropic inflammatory cytokine playing a vital role in the pathophysiological mechanism of various processes [50]. As an effective commanding profibrogenic mediator [51], TGF-β1 mediates HSC’s activation and promotes excessive ECM production through targeting a wide spectrum of downstream proteins specially SMADs [52]. Therefore, TGF-β1 has been emerged as a putative therapeutic target for the control of liver fibrosis. Our findings, as documented by the immune-histochemistry and in parallel to qRT-PCR mRNA results, show that hepatic expression of TGF-β1 was downregulated in the D-carvone treated group, as compared to the fibrosis model. This effect was associated with a decrease in the protein expression of SMAD3. Our results concur with previous studies [53,54], where they established a correlation between activation of TGF-β1/SMAD3 pathway and fibrogenesis in different animal models of hepatic fibrosis. The beneficial effect of D-carvone on regulation of TGF-β1/SMAD3 pathway entailed the modulation of HSCs activation and fibrogenesis, verified by decreasing α-SMA expression and collagen deposition, as compared to the CCl_4_ intoxication. Accordingly, it was found that elevation of α-SMA protein expression in liver can be owed to the activated TGF-β1pathway both in vivo and in vitro [55]. Apart from its SMAD-dependent profibrogenic role, activated TGF-β1 extends its effect to activate MAPK, that in turn stimulates the NF-κB transcription factor which promotes inflammation and fibrosis [53,56].

Although the mechanism by which D-carvone suppresses the TGF-β1/SMAD3 pathway is unknown, it is worth noting that D-carvone has been shown to inhibit Janus kinase (JAK)/signal transducer and activator of transcription 3 (STAT3) signaling pathway, which has been reported to be implicated in the pathophysiology of fibrosis [23]. Ongoing hepatic injury and inflammation result in persistent activation of JAK/STAT signaling in response to inflammatory cytokines, most notably to interleukin (IL)-6 family members. A cross talk between JAK/STAT pathway and SMAD pathway, via a transcriptional cooperation, has been previously suggested to mediate the roles of TGF-β in fibrogenesis [23]. D-carvone inhibits the phosphorylation of the JAK-STAT3 signaling molecules in a dose dependent manner [15].

Furthermore, previous studies revealed a close relation between inflammasomes’ activation and the fibrogenesis process, whereby the persistent activation of the nucleotide-binding oligomerization the domain-like receptors (NLRs) and the transmembrane toll-like receptors (TLRs) inflammasome complex mediates the production of IL-1β and IL-18 proinflammatory cytokines that increase TGF-β1 expression and subsequently trigger epithelial-to-mesenchymal transition for myofibroblasts formation, which are major contributors to aggravating the development of fibrotic lesions [57,58]. A recent study describes a novel mechanism by which D-carvone protects against cerebral ischemia/reperfusion-induced inflammatory response by suppressing the TLR4/NLRP3 cascade [24]. Thereby, the observed downregulation of TGF-β1 by D-carvone can also be explained by its suppressing effect on TLR4/NLRP3 cascade.

Several studies have reported that Nrf2 activators dramatically inhibit liver fibrosis, suggesting the antifibrotic effect of Nrf2. In liver, Nrf2 activation was found to inhibit fibrogenesis process by promoting fibroblast differentiation and inhibiting TGF-β1/SMAD-dependent HSCs activation [59]. Therefore, activation of Nrf2 by D-carvone and its derivatives could contribute to the observed reduction in the TGF-β1 expression in the current study.

MMP9 is a gelatinase enzyme belongs to MMP family of zinc metallo-endopeptidases. MMP9 is implicated in the fibrolytic degradation of specific ECM proteins such as fibronectin and type IV collagen [60,61]. In addition, MMP-9 contributes to resolution of liver fibrosis by promoting HSCs apoptosis [62]. From the data presented, chronic CCl_4_ intoxication induced a marked downregulation of MMP9 gene expression in the liver, which could favor the accumulation of ECM [63], whereas, D-carvone effectively restored MMP9 expression level.

Previous reports have suggested a crosstalk between TGF-β1 and MMP-9, whereas MMP-9 inhibition in the late stage of fibrosis seems to be mediated by TGF-β1 [64,65]. This evidence is in agreement with our findings. We found that the increased hepatic expression of TGF-β1 was accompanied with downregulation of MMP9 in CCl_4_ hepatic stellate cells group. D-carvone administration significantly diminished TGF-β1 and enhanced MMP-9 expression compared to the model rats. A possible explanation of these observations is that the modulatory effects of D-carvone on TGF-β and MMP9 rely on its anti-inflammatory effects [24,28], to coincide with the findings of previous reports [66,67].

Overall, D-carvone modulated many processes in the CCl_4_-induced fibrotic liver, including the oxidative stress, collagen deposition, and expression of pro-fibrogenic mediators including TGF-β1, SMAD3, α-SMA, and MMP9. The obtained findings, together with the safety profile reported by previous studies [9,13,14,15,16,17], suggest that D-carvone could serve as a promising anti-fibrotic therapeutic agent, since several hepatoprotective agents that had been tested in animal models of ongoing hepatic damage are proven to be successfully translated into clinically useful therapeutics [68,69,70,71]. Therefore, current findings could prove that these are highly relevant data and can be translated to the clinic.

Although in vivo studies using animal models have several limitations, including expensive experiments, interindividual variation, and the need for a large number of animals and sample size, they provide the advantage of having the highest degree of correlation with what occurs within the complexity of biological systems; they also allow variable biochemical and histological assessments to be performed [72]. In addition, CCl_4_ is a standard model of hepatic fibrosis. However, liver fibrosis induced by CCl_4_ is reversible after withdrawal of CCl_4_ treatment, thus, CCl_4_ was concurrently given with D-carvone treatment. Other models with a lower degree of regression and in vitro studies to investigate the direct effects of D-carvone on hepatic stellate cells should be further conducted.

The current study addressed some shortcomings. First, our study did not adopt a time course of D-carvone treatment or dose–response calculation that could be useful for clinical studies. Second, this study is missing the animal body weight, liver weight, and age relationship at the end of the experiment. In addition, protein expression of the key molecules involved in mediating the modulatory effects of D-carvone on TGF-ß 1/SMAD3 signaling pathway also needs further study.

## 4. Materials and Methods

### 4.1. Chemicals

D-carvone (S-5-Isopropenyl-2-methyl-2-cyclohexenone; ≥96%) and CCl_4_ (99.8%) were purchased from Sigma-Aldrich Corp., St. Louis, MO, USA. The rest of the chemicals and reagents were of analytical grade and were purchased from Sigma Aldrich, USA.

### 4.2. Animals

Male albino Wistar rats (150–170 g) were obtained from the animal house of Research Institute of Ophthalmology (Giza, Egypt). Prior to the experiment, the rats were acclimatized in groups of eight animals/cage, humidified and maintained under standard conditions with adequate water and food (standard chow diet). The experiment was conducted for eight weeks during which rats were treated according to the ethical guidelines as approved by the Institutional Animal Care and Use Committee, Cairo University (ethical no.: CU-II-F-1-18).

### 4.3. Experimental Design

**Figure 10:**
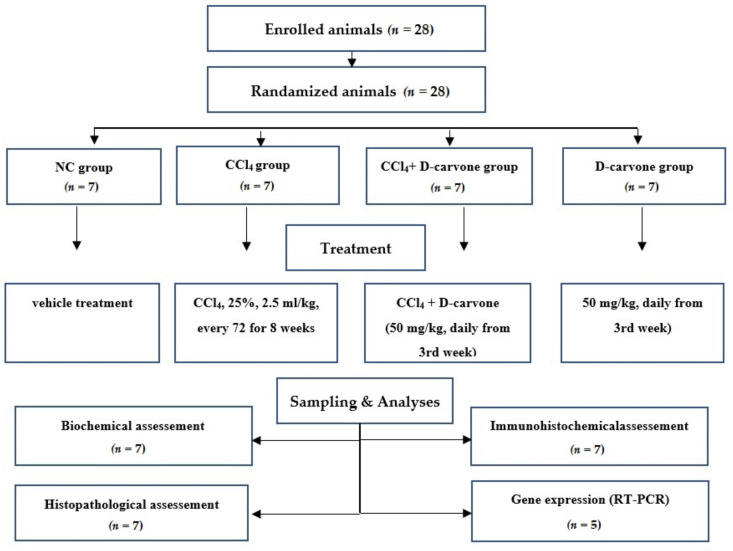
Flow chart of the experimental protocol. A total of 28 rats were enrolled in this experiment and underwent randomization. Rats were assigned to 4 groups. The total number of samples was 7 for biochemical, histological, and immunohistochemical examinations and 5 for RT-PCR gene expression analysis.

As presented in Figure 10, animals were randomized into four groups (*n* = 7); the normal control group (NC) received vehicle treatment, the CCl_4_ group endured CCl_4_ in corn oil (25% *v*/*v*, 2.5 mL/kg) every 72 h via intraperitoneal injection for 8 weeks to induce liver fibrosis [45], CCl_4_+ D-carvone group was given D-carvone in corn oil (50 mg/kg b.w.) via intragastric daily from the third week of CCl_4_ administration, and the D-carvone group received D-carvone only. The dose of D-carvone was selected based on the previous study of Muruganathan and Srinivasan [36].

Next, 24 h after the last treatment, animals were anesthetized to collect blood samples for serum separation, then to obtain liver tissues. Immediately after euthanization, livers were excised, washed with saline, and either snap-frozen in liquid nitrogen and stored at −80 °C for biochemical and molecular analyses or processed for histopathological analysis.

### 4.4. Serum and Tissue Biomarkers

Liver function indices (ALT and AST) were assessed in serum. Liver homogenate (10%) in 0.1 M cold phosphate buffer saline (pH 7.4) was prepared to assess malondialdehyde (MDA) as a lipid peroxidation (LPO) marker, reduced glutathione (GSH) and superoxide dismutase (SOD) as antioxidant markers, and total antioxidant capacity (TAC). All parameters were determined using the corresponding test commercial kits (Bio diagnostic, Cairo, Egypt).

### 4.5. Histopathological Assessment

Fresh liver specimens (*n* = 7/group) were immediately fixed in 10% neutral buffered formalin for 48 h, dehydrated, and processed to obtain 4–5 μm paraffin embedded sections. Sections were de-waxed in xylene, rehydrated in ethanol, and stained with standard hematoxylin-eosin (H&E) or Masson trichrome (MT) Staining [73]. Image analysis was performed by Image analysis software (Image J, version 1.46a, NIH, Bethesda, MD, USA). The stage of liver fibrosis was graded based on the histological scoring system described by Ishak et al. [74], as follows: 0 = no fibrosis, 1 = fibrous expansion of some portal areas, 2 = the most portal areas were expanded by collagen fibers, 3 = portal fibrosis with infrequent portal-to-portal bridging, 4 = marked portal to portal bridging and portal to central bridging, 5 = marked portal to central bridging and central to central bridging with occasional nodules, 6 = definite cirrhosis.

### 4.6. Quantitative Real-Time PCR Analysis (qRT-PCR)

Total RNA was extracted from liver samples using TRIzol™ Reagent (Qiagen, Hilden, Germany). One µg of total RNA was used as a template for the first strand cDNA synthesis using SuperScript IV VILO reverse transcriptase kit (Invitrogen, Waltham, MA, USA). qPCR was carried out using StepOnePlus Real Time PCR Kits (Applied Biosystems, Foster City, CA, USA) and oligonucleotide primers specific for the target genes, as listed in Table 1. The relative mRNA expression was calculated according to 2^−∆∆Ct^ method [75]. Triplicate analysis for each cDNA was conducted with a minus-template negative control. β-actin gene (endogenous control) was amplified during each RT-PCR run to normalize the Ct values of the target genes. Results are expressed as fold change relative to normal control [76].

### 4.7. Immunohistochemical Assessment

Immunohistochemical assessment (IHC) was performed on paraffin sections to evaluate α-SMA TGF-β1 and SMAD3 protein expression according to standard methods in routine pathology, as described by [80]. The antigen retrieval was performed according to [81]. Tissue sections from different groups were incubated with one of the following primary antibodies: anti-α-SMA rabbit polyclonal antibody with dilution 1:200 (ab5694; Abcam, Cambridge, UK), anti-TGF-β1 rabbit polyclonal antibody with concentration of 20 µg/mL (ab92486; Abcam, Cambridge, UK), and anti-SMAD3 rabbit polyclonal antibody with dilution 1:200 (ab28379; Abcam, Cambridge, UK) overnight in a humidified chamber. The tissue sections were incubated with secondary antibody, Goat anti-rabbit IgG H&L (HRP) (ab205718; Abcam, Cambridge, UK). For visualization of the reaction, the tissue sections were incubated with 3,3′-diaminobenzidine tetrahydrochloride (DAB, Sigma) and counterstained with Mayer’s hematoxylin. Image analysis software (Image J, version 1.46a, NIH, Bethesda, MD, USA) was used to measure the integrated optical density of α-SMA, TGF-β1, and SMAD3 positive expression [82].

### 4.8. Statistical Analysis

All values are expressed as mean ± standard deviation (SD). GraphPad Prism 5 Software (GraphPad, San Diego, CA, USA) was adopted for data analysis. One-way analysis of variance (ANOVA) followed by Tukey’s test were employed for comparisons among groups, and *p*-value ≤ 0.01 was regarded as statistical significance [83].

## 5. Conclusions

The current study is the first report to highlight the protective potential of the monoterpene D-carvone against hepatic fibrosis. D-carvone exerts significant hepatoprotective and anti-fibrotic effects that may be, at least in part, due to its antioxidant and anti-inflammatory activities. Based on the current results, this study speculated the involvement of TGF-β1/SMAD3 pathway mediating this anti-fibrotic activity. Our data suggest that D-carvone provides an accessible source of natural antioxidants that could provide benefit as a therapeutic agent in controlling liver fibrosis. However, further comprehensive mechanistic studies are warranted to elucidate other mechanisms pertaining to the anti-fibrotic activity of D-carvone before proceeding to human clinical trials.

## Figures and Tables

**Table 1:** Primer sequence for q-RT-PCR.

Gene	Forward (5′-3′)	Reverse (5′-3′)	Accession #	Ref.
TGFß1	GGACTCTCCACCTGCAAGAC	CTCTGCAGGCGCAGCTCTG	NM_021578.2	[77]
MMP9	CACTGTAACTGGGGGCAACT	CACTTCTTGTCAGCGTCGAA	NM_031055.2	[78]
ß-actin	ATGGTGGGTATGGGTCAG	CAATGCCGTGTTCAATGG	XM_034489257.1	[79]

TGF-β1, transforming growth factor-β1; MMP9, matrix metalloprotease 9.

## Data Availability

Data available upon request.
